# Cross‐talk between CFTR and sphingolipids in cystic fibrosis

**DOI:** 10.1002/2211-5463.13660

**Published:** 2023-06-22

**Authors:** Dorina Dobi, Nicoletta Loberto, Rosaria Bassi, Anna Pistocchi, Giulia Lunghi, Anna Tamanini, Massimo Aureli

**Affiliations:** ^1^ Department of Medical Biotechnology and Translational Medicine University of Milan Italy; ^2^ Section of Clinical Biochemistry, Department of Neurosciences, Biomedicine and Movement University of Verona Italy

**Keywords:** CFTR, cystic fibrosis, gangliosides, metabolism, plasma membrane, sphingolipids

## Abstract

Cystic fibrosis (CF) is the most common inherited, life‐limiting disorder in Caucasian populations. It is caused by mutations in the gene encoding the cystic fibrosis transmembrane conductance regulator (CFTR), which lead to an impairment of protein expression and/or function. CFTR is a chloride/bicarbonate channel expressed at the apical surface of epithelial cells of different organs. Nowadays, more than 2100 CFTR genetic variants have been described, but not all of them cause CF. However, around 80–85% of the patients worldwide are characterized by the presence, at least in one allele, of the mutation F508del. CFTR mutations cause aberrant hydration and secretion of mucus in hollow organs. In the lungs, this condition favors bacterial colonization, allowing the development of chronic infections that lead to the onset of the CF lung disease, which is the main cause of death in patients. In recent years, evidence has reported that CFTR loss of function is responsible for alterations in a particular class of bioactive lipids, called sphingolipids (SL). SL are ubiquitously present in eukaryotic cells and are mainly asymmetrically located within the external leaflet of the plasma membrane, where they organize specific platforms capable of segregating a selected number of proteins. CFTR is associated with these platforms that are fundamental for its functioning. Considering the importance of SL in CFTR homeostasis, we attempt here to provide a critical overview of the literature to determine the role of these lipids in channel stability and activity, and whether their modulation in CF could be a target for new therapeutic approaches.

## Graphical abstract

Several lines of evidence shown that in cystic fibrosis the lack of the cystic fibrosis transmembrane conductance regulator (CFTR) is accompanied by changes in the plasma membrane composition of a bioactive class of lipids known as sphingolipids. Here, we depict a general overview of the literature related to the impact of the modifications of the sphingolipid pattern on the stability of normal and mutated CFTR.

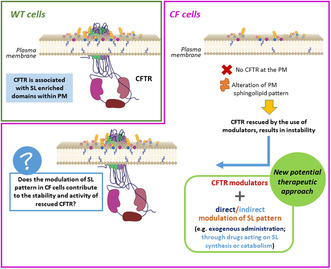

**Abbreviations**
ALI: air–liquid interface
cAMP: cyclic adenosine monophosphate
Cer: ceramide
Cer‐1‐P: ceramide‐1‐phosphate
CerPE: ceramide phosphoethanolamine
CF: cystic fibrosis
CFTR: cystic fibrosis transmembrane conductance regulator
GlcCer: glucosylceramide
GSL: glycosphingolipids
HBE: human bronchial epithelial
LacCer: lactosylceramide
MSCs: mesenchymal stem cells
NHERF1: Na^+^/H^+^ exchanger regulatory factor
PM: plasma membrane
RD: regulatory domain
rhAC: recombinant human acid ceramidase
SL: sphingolipids
SM: sphingomyelin
TFEB: transcription factor EB
WT: wild‐type

Sphingolipids (SL) are bioactive lipids ubiquitously present in the plasma membrane (PM) of all eukaryotic cells. They consist of a hydrophobic backbone that is inserted in the core of the membrane and a hydrophilic head that protrudes into the extracellular milieu [1]. This structure determines that SLs are not randomly distributed in the PM, but are mainly asymmetrically associated with its external leaflet. Due to their peculiar physicochemical properties, they can organize specific membrane domains, called lipid rafts, which, together with cholesterol, are able to sequester specific proteins and regulate their function [2, 3, 4]. The definition of the SL pattern is the result of a complex metabolic machinery. Every cell is characterized by a specific SL composition that depends on its function and physiological state, and variations in the SL are often associated with several diseases. In recent years, alterations in the SL pattern of cells were found in cystic fibrosis (CF). CF is the most common life‐threatening hereditary disease among Caucasians, and it is caused by mutations in the gene encoding for a chloride‐conducting transmembrane channel called cystic fibrosis transmembrane conductance regulator (CFTR), which lead to an impairment of CFTR protein expression and/or function. The ion channel is expressed at the apical surface of epithelial cells. CFTR loss of function is responsible for the aberrant dehydration of mucus with the consequent colonization of pathogens and onset of chronic infections. In this context, several studies have reported how changes in the SL composition could affect the inflammatory response in CF, as well as the capability to clear bacteria. In addition, more than 160 papers (PubMed sources) highlight the involvement of SL in CF pathology. Based on the emerging importance of these lipids in CF, here we provide a critical summary of the state of the art on this aspect of the pathology. In particular, we present the pathology and the general overview related to SL metabolism, focusing on the SL changes that occur in CF. Moreover, we try to dissect whether SL could be considered as molecules belonging to the CFTR interactome and whether their modulation could represent an adjuvant approach to therapeutic strategies currently available.

## Cystic fibrosis

Cystic fibrosis is the most common autosomal recessive disease among Caucasians, affecting approximately 1 in 2500–4000 newborns, and is caused by loss of function mutations in the gene encoding the CFTR protein [5]. CFTR is a large multidomain protein belonging to the ABC subfamily, composed of 1480 amino acid residues arranged in two membrane‐spanning domains (MSD1, 2), two nucleotide (ATP)‐binding domains (NBD1, 2), and a regulatory domain (RD) with multiple phosphorylation sites. It is a cyclic adenosine monophosphate (cAMP)‐activated chloride/bicarbonate channel expressed at the apical surface of epithelial cells of different organs, such as lungs, pancreas, intestines, and sex organs [6]. In CF, mutations in the *CFTR* gene lead to reduced expression of the functional channel resulting in an impaired chloride ion transport of secretory epithelial cells. CF can either have a severe course with fast progression of symptoms or a milder course with less lung deterioration. Although CF affects multiple tissues, the most severe manifestations occur in the respiratory system. The defective ion secretion causes insufficient osmotic pressure, increased absorption of water followed by dehydration of the airway surface liquid, and diminished mucociliary clearance. Consequently, mucus accumulates on the bronchial surface of epithelial cells, promoting tissue destruction, inflammation, and recurrent bacterial infections with chronic colonization, mainly by *Pseudomonas aeruginosa*, representing the main cause of morbidity and mortality in CF patients. Nevertheless, complications can occur in nearly every organ including, among others, liver disease, CF‐related diabetes, nasal polyps, intestinal obstructions, and allergic bronchopulmonary aspergillosis [7, 8].

Although more than 2100 mutations have been described in the *CFTR* gene with a wide range of biological and functional phenotypes, not all of them cause CF (https://cftr2.org/, accessed on 22 December 2022). Currently, the mutations responsible for the development of CF are classified into seven different classes based on the effect generated on the channel. Class I, II, III, and VII mutations are associated with no residual CFTR function, and patients with these kinds of mutations show a severe phenotype characterized by lung and pancreatic function impairment. On the contrary, patients with mutations belonging to class IV, V, and VI show some residual CFTR function with mild lung disease and pancreatic sufficiency. The most common mutation accountable for the onset of CF is the deletion of a phenylalanine at position 508 (F508del) within the nucleotide‐binding domain 1 (NBD1), which causes misfolding and retention of the CFTR in the ER with consequent premature degradation. Even, the small percentage of the immature protein able to reach the PM is not stable and rapidly internalized and degraded [9, 10].

Although significant therapeutic improvements have extended the life expectancy of CF patients, to date there is no cure for this disease. The most promising therapeutic strategies are based on the use of CFTR modulators. Among them, there are correctors, capable of improving the biosynthesis and trafficking of CFTR towards the PM, such as VX‐809 (Lumacaftor), VX‐661 (Tezacaftor), and VX‐445 (Elexacaftor), and potentiators, like VX‐770 (Ivacaftor), enhancing the function of the mutated channel once it has reached the PM. Until now, VX‐770 is the only potentiator that has been approved as a monotherapy treatment for CF patients with gating mutations. Otherwise, therapeutic approaches for CF patients carrying the F508del mutation are based on the simultaneous administration of correctors and potentiators [11]. Nowadays, we are in a new era for patients carrying this mutation, at least in one allele, because they can be treated with the triple combination VX‐661, VX‐445, and VX‐770, called Kaftrio. The follow‐up of patients treated with this new formulation indicates an important improvement in the clinical symptoms and, of course, life expectancy. Despite this great medicinal option, it is important to consider that for 20–30% of CF patients, a therapeutic approach such as Kaftrio is not available, and for this reason, the necessity of developing new modulators and potentiators is still an important challenge in CF.

## Sphingolipids

**Fig. 1:**
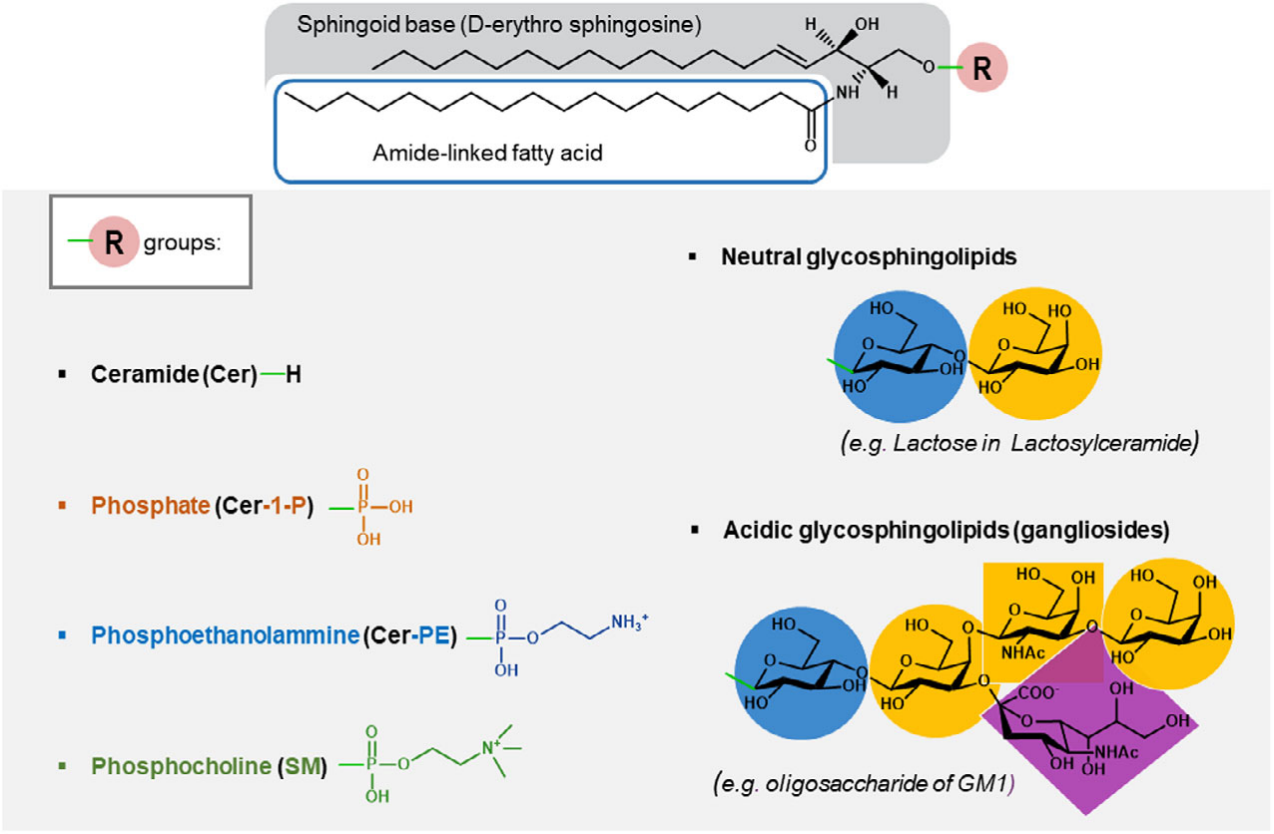
Structure of the main representative sphingolipid types.

Sphingolipids are a complex class of lipids found in all eukaryotic cells where they serve as structural components of membranes [12]. SLs are predominantly associated with the external leaflet of the PM, where they are restricted in specific membrane areas, known as lipid rafts, organizing platforms where specific proteins involved in the signaling mediated across the PM are recruited [13]. They are defined as a category by the presence of sphingosine (Sph), a long chain base to which a fatty acid is linked by an amidic bond‐giving ceramide (Cer), the basic building block of all SLs. The most frequent sphingoid bases occurring in mammalian cell SLs are C‐18 and C‐20 sphingosines with a double bond at the C4‐C5 position followed by C‐18 and C‐20 sphinganines lacking the C4‐C5 double bond and present in very small amounts. The fatty acid linked to Sph can significantly vary in chain length and in grade of saturation and/hydroxylation depending on the tissue and the cell type, with palmitic acid (C16:0) and stearic acid (C:18:0) as the predominant species. The primary hydroxyl group of Cer may be linked to phosphocholine, giving rise to sphingomyelin (SM), the main phosphosphingolipid with ceramide‐1‐phosphate (Cer‐1‐P), ceramide phosphoethanolomine (CerPE) and the corresponding de‐acylated forms or lysoderivatives. Glycosphingolipids (GSLs) represent the other major subclass of SLs characterized by a glycan moiety β‐glycosidally linked to Cer. The saccharide structure may be a single monosaccharide unit, such as glucose or galactose, yielding the simplest GSL, glucosylceramide (GlcCer), and galactosylceramide, which are neutral GSL as lactosylceramide (LacCer) derived from GlcCer by the addition of galactose, or an oligosaccharide chain containing up to 20 monosaccharide units giving rise to more complex GSLs. The presence of one or more residues of *N*‐acetlyneuraminic acid (Neu5Ac) confers a negative charge to the glycan moiety which determines the structures of gangliosides, the main subclass of acidic GSLs. The structural differences in the headgroup and in the hydrophobic lipid backbone of SLs confer unique features to each of these amphipathic molecules, thus explaining their multiple functions as modulators of membrane proteins (enzymes, transporters, receptors, and ion channels) and their different roles in fundamental cell processes [14]. The chemical features of some of the most representative SLs are shown in Fig. 1.

## CFTR deficiency and its correlation with sphingolipids

For many years, research in CF was focused on CFTR‐centered studies and on how different mutations affect its interactome protein composition. However, CFTR loss of function itself can influence other factors like inflammation and the metabolism of other important cellular components, such as lipids. Indeed, it is well known that the inactivity of the channel, as well as bacterial infections and typical characteristics of CF patients, is also responsible for alterations in the lipid composition of the PM, especially the sphingolipids.

In CF, most studies are related to alterations in the Cer content in various *in vitro* and *in vivo* models of the pathology, as well as in samples derived from patients. It is a general consensus among the scientific community that elevated levels of some ceramides in the PM promote the development of an inflammatory phenotype that contributes to the onset of the CF lung disease [15, 16, 17, 18, 19, 20, 21].

The increased Cer content was classically attributed to bacterial infections. Indeed, several bacteria dispose the sphingomyelinase enzyme as a common virulence factor, which converts SM into Cer directly at the cell surface. In addition, Cer can be generated by the hydrolysis of complex SL mediated by several enzymes associated with the cell surface [22].

In fact, it has also been demonstrated that bacterial infections, such as *P. aeruginosa*, through different mechanisms, are able to increase the activity of two host enzymes that exert their function directly at the PM, namely the neutral sphingomyelinase and the nonlysosomal β‐glucosylceramidase. This last enzyme is responsible for the catabolism of glucosylceramide into Cer and glucose at the PM [15, 23].

Brodlie *et al*. [24] found that Cer accumulates in the trachea and epithelial cells of large and small bronchi of various CFTR‐deficient mice before any infections and in lungs from CF patients. Moreover, increased Cer levels were described in macrophages in both CF mice models and patients [25].

Unbiased metabolomic approach also identified an increase in Cer content in the sputum of CF patients after exacerbation of the disease [26], suggesting a possible role of this lipid as a biomarker in determining the different stages of the disease.

Alterations in ceramide content were likewise observed in an *in vitro* cellular model of CF mesenchymal stem cells (MSCs) represented by control human lung MSCs, where CFTR activity is blocked using a specific CFTR inhibitor (inh172). CF‐MSC cells are characterized by a reduction in the total amount of SM, followed by an increase in the Cer content when compared with MSCs with a functional CFTR. In addition, the analyses performed on the extracellular vesicles released by the CF‐MSCs revealed an elevated content of Cer. Interestingly, these vesicles showed a decreased anti‐inflammatory effect with respect to those isolated from MSCs expressing the wild‐type (WT) form of the channel [27].

Furthermore, modifications in the content of molecular species of Cer were also observed. Especially, a significant increase in C16, C18, and C20‐Cer content was found with a concomitant reduction in the prevalent and anti‐inflammatory molecular species, the C24‐Cer [28, 29]. Although the analysis of molecular species of ceramide is often overlooked, they are of crucial importance. The presence of a long fatty acid in the acyl chain increases the formation of interdigitation bonds with the hydrophobic chain of lipids belonging to the inner layer of the PM. This condition not only changes the local thickness of the membrane, making it thinner, but could also represent the driving force behind the organization of specific signaling platforms at the PM [30].

Several studies are likewise related to the investigation of blood SL content as a predictive marker of disease stage. Unfortunately, due to conflicting data in the literature, probably due to different handling of the samples, it is difficult to evaluate whether blood SL levels could be considered as true biomarkers. Nevertheless, because of their importance in the disease, further studies need to be performed to address this notable gap [31].

It is reasonable to hypothesize that the reduced catabolism of Cer in the lungs of CF models, reported by Grassmè *et al*. [21], leads to a low production of sphingosine, an important bactericidal molecule [32, 33, 34]. However, this is only speculation because more than 90% of sphingosine is obtained in the lysosomes by Cer catabolism and reused in the *de novo* biosynthetic pathway of SL.

To explain the reduced levels of sphingosine, it is reasonable to speculate that CF cells are characterized by an alteration in the activity or expression of the neutral ceramidase, the enzyme responsible for Cer catabolism at the PM.

Lipidomic analysis performed on human IB3‐1 immortalized cell lines, which are epithelial cells carrying biallelic F508del mutation, showed that beyond the accumulation of Cer there is also an increased content of GSL. GSL are a particular class of SL carrying a hydrophilic moiety composed of different sugars. GSL biosynthesis occurs at the cytosolic side of early Golgi membranes by the action of different glycosyltransferases that transfer a specific carbohydrate from the sugar nucleotide (UDP‐Glucose, UDP‐Galactose) to a specific acceptor, such as ceramide or other sugars. On the contrary, GSL catabolism occurs mainly in lysosomes and consists in the remodeling of their hydrophilic head by the sequential action of lysosomal hydrolases [35, 36].

The first step of their biosynthesis starts with the glycosylation of Cer to GlcCer. Afterward, the neo‐synthetized GlcCer can either reach the PM or it can be translocated to the luminal side of the Golgi, where it is further glycosylated by different glycosyltransferases leading to the formation of more complex GSL [37]. For instance, the addition of a galactose residue, by the action of the galactosyltransferase enzyme, results in the formation of the LacCer which is the precursor of all complex GSL. One of the main branches of LacCer metabolism is the formation of gangliosides, a class of GSL characterized by the presence of one or more sialic acid residues on the oligosaccharide chain. GM3 synthase or sialyl‐transferase is responsible for the sialylation of LacCer to form GM3. Other metabolites in this pathway are formed through similar reactions; for instance, GM3 is converted to GM2 by GM2 synthase, which transfers an *N*‐acetylgalactosamine to GM3, and then, GM2 is converted to GM1 by GM1 synthase [38].

In particular, accumulation of GlcCer, LacCer, and monosialoganglioside GM3 was found in CF cell lines [39], and increased expression of GlcCer has been associated with tissue damage and exacerbation of inflammatory responses, typical features in CF [40, 41, 42].

Even if it is less studied in the contest of CF, the ganglioside pattern is also impaired. A reduction in ganglioside GM1 levels was observed both in human primary bronchial epithelial (HBE) cells carrying biallelic F508del mutation in the *CFTR* gene differentiated at air–liquid interface (ALI) and in the CFBE41o‐cell line overexpressing the same mutated form of the channel [43]. Moreover, GM1 lacking the sialic acid (asialo‐GM1) is found increased in the 9HTEo‐tracheal epithelial cell line overexpressing the RD of CFTR. Interestingly, one explanation for the reduction in the content of GM1 could be its increased conversion into asialo‐GM1; however, experiments have not been conducted to determine the mechanism of either the decrease in GM1 or the increase in asialo‐GM1 levels [44]. Importantly, the reduction in GM1 seemed to cause delayed wound repair in Calu‐3 cells, whereas restoring GM1 recovered this delay [8].

Recent data showed that the mutation F508del also alters SL composition during *in vitro* differentiation of HBE cells at ALI. This model resembles an airway epithelium, since during differentiation several cell populations are formed, such as ciliated, goblet, club, and basal cells. The different stages of differentiation *in vitro*, represented by the proliferative stage, the early differentiation stage, and the fully differentiated stage, resemble the physiological process that occurs during the *in vivo* differentiation of lung stem cells [45]. Interestingly, analyses of SL levels in HBE cells from WT‐CFTR patients showed that during differentiation, there is an increase in Cer and globotriaosylceramide, while GlcCer and ganglioside levels decrease. On the contrary, HBE cells carrying biallelic F508del mutation in the *CFTR* gene are characterized by a further increase in the Cer and GlcCer content and by a more marked reduction in the ganglioside GM1 levels at all stages of differentiation, compared with WT‐CFTR cells [45]. Not only the content, but also the subcellular localization of lipids in fully differentiated cells is different between WT and CF HBE. The apical membrane showed an enrichment of SL in comparison with the levels found in the basolateral membrane of differentiated epithelia. Interestingly, the increase in Cer and GlcCer content is mainly localized at the apical membrane of CF HBE cells [45]. Due to the specific chemical–physical properties of these SL, this evidence has led to speculation that the apical membrane of CF cells is characterized by a more rigid structure that could compromise its function [46, 47]. Moreover, high levels of Cer and GlcCer support the hypothesis that pro‐inflammatory stimuli are triggered directly from the luminal side of cells exposed to possible infections and to cells of the immune system that are recruited to the site of infection. Interestingly, in CF cells the increased content of Cer seems to be due to the increased activity of the PM associated β‐glucocerebrosidase and nonlysosomal β‐glucosylceramidase, both enzymes involved in the catabolism of the GlcCer at the PM (1.4‐ and 1.5‐fold, respectively), compared with WT‐CFTR cells. On the contrary, the augmented level of GlcCer lets us speculate that there may be an increase in the biosynthetic pathway [31, 45]. One of the new challenges following the introduction of CFTR modulators is the study of their effect on the SL pattern. Emerging data showed that the use of the triple formulation Kaftrio seems to rebalance the composition of SL; however, confirmatory studies are needed to further understand the effects of highly effective CFTR modulators on the CF lipidome [31].

## Sphingolipids as interactors of CFTR

It is well established that there is a fundamental cross‐talk between PM proteins and the surrounding lipids. There is a tendency to study the two elements separately, but this could lead to misinterpretation of the results as: (a) aberrant expression of a protein, like in the case of CFTR, can affect the lipid composition, and modifications of the SL pattern may affect protein expression and function, (b) fine lipid–protein interactions, especially at the PM, are essential for the protein function and thus for ensuring the homeostasis of the cells. Moreover, significant alterations in the lipid composition, such as in case of CF bronchial epithelial cells, can determine changes in the structure of the PM regarding thickness, curvature, and flexibility. These parameters are fundamental for protein stability and function. A membrane fraction that is too thin compared with the transmembrane domain of the protein leads to changes in protein structure to minimize the exposure of the hydrophobic domain to the extracellular or intracellular hydrophilic environment [48, 49]. Modifications in the curvature as well as in the flexibility of the membrane also change the lateral pressure exerted by the lipid–lipid interactions. This aspect is of great importance for proteins with channel activity. Indeed, during its opening, the channel changes its conformation, increasing the volume occupied in the membrane. If the lateral pressure of the lipids is not adequate, this cannot occur, and the channel remains closed [50].

On the contrary, it is possible that changes in the lipid composition do not have dramatic consequences on the membrane architecture but may more subtly determine modifications in lipid–protein interactions. Proteins and lipids can interact in different ways, like direct allosteric interaction, interdigitation, and through the organization of membrane platforms characterized by a different fluidity with respect to the rest of the membrane. First, allosteric interaction means that lipids interact with proteins and the binding induces modifications in the protein structure that can have positive or negative effects on its activity. A demonstration is represented by the allosteric regulation of cholesterol on the potassium channel 2 of the PM. Indeed, cholesterol is a negative allosteric regulator since its binding reduces the activity of this channel [51, 52, 53]. Second, interdigitation is normally mediated by the hydrophobic core of the lipid and that of the protein (acyl chain or transmembrane domain). As an example, Iwabuchi and coworkers demonstrated an interaction between the hydrophobic portion of the long chain of LacCer and the nonreceptor tyrosine kinase Lyn. They suggested that the protrusion of the hydrophobic chains of LacCer into the cytoplasmic membrane leaflet could be so pronounced that it allows direct van der Waals interactions between the acyl chains of Lyn and LacCer. In neutrophils, this interaction is fundamental for the activation of Lyn and consequently for the promotion of superoxide generation and cell migration [30].

Finally, due to their physicochemical properties, sphingolipids are nitrogen bond donors and acceptors in the PM. This feature enables their self‐segregation with the establishment of a tight net of hydrogen bonds, which, together with cholesterol, saturated glycerophospholipids, and a selected number of proteins, leads to the organization of lipids rafts [54].

**Fig. 2:**
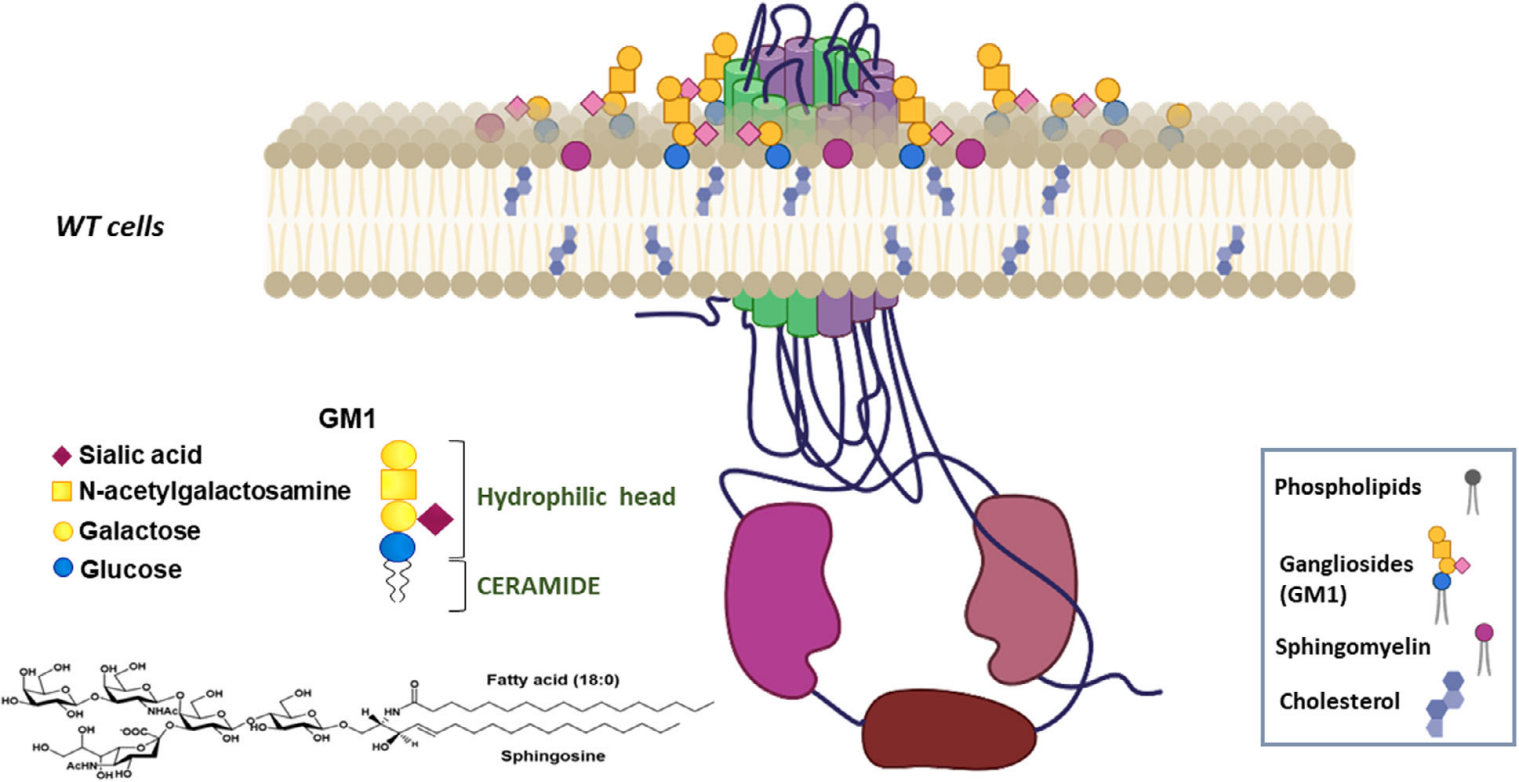
Schematic representation of WT‐CFTR associated with lipids rafts.

There are two types of channels located in the apical cell membrane: the ones freely floating in the lipid bilayer, and the others confined to lipid rafts, such as CFTR. It is important to note that the association of CFTR with lipid rafts strongly depends on the type of cells considered. Indeed, in HBE cells cultured *in vitro*, the proportion of CFTR protein confined to lipid rafts represents around 25% of the total amount, whereas in Calu‐3 cells, this population reaches up to 50% [55, 56]. Nevertheless, in HBE cells, CFTR belongs to specific lipid rafts in which it can interact with ganglioside GM1 and cholesterol. This localization is fundamental for the recruitment of the scaffolding proteins NHERF1 and Ezrin, stabilizing the channel and enabling its activity [43, 55, 57] (Fig. 2). In addition, in murine tracheal epithelial cells, CFTR is found to localize in GM1‐positive lipid rafts and its localization is not affected by *P. aeruginosa* infections [58].

Interestingly, activation of the tumor necrosis factor receptor and inhibition of Src kinase enhances the association of these signaling molecules with lipid raft components. This process is accompanied by the recruitment of CFTR to detergent‐resistant membrane fractions, suggesting that CFTR may partition into microdomains during inflammation [59].

**Fig. 3:**
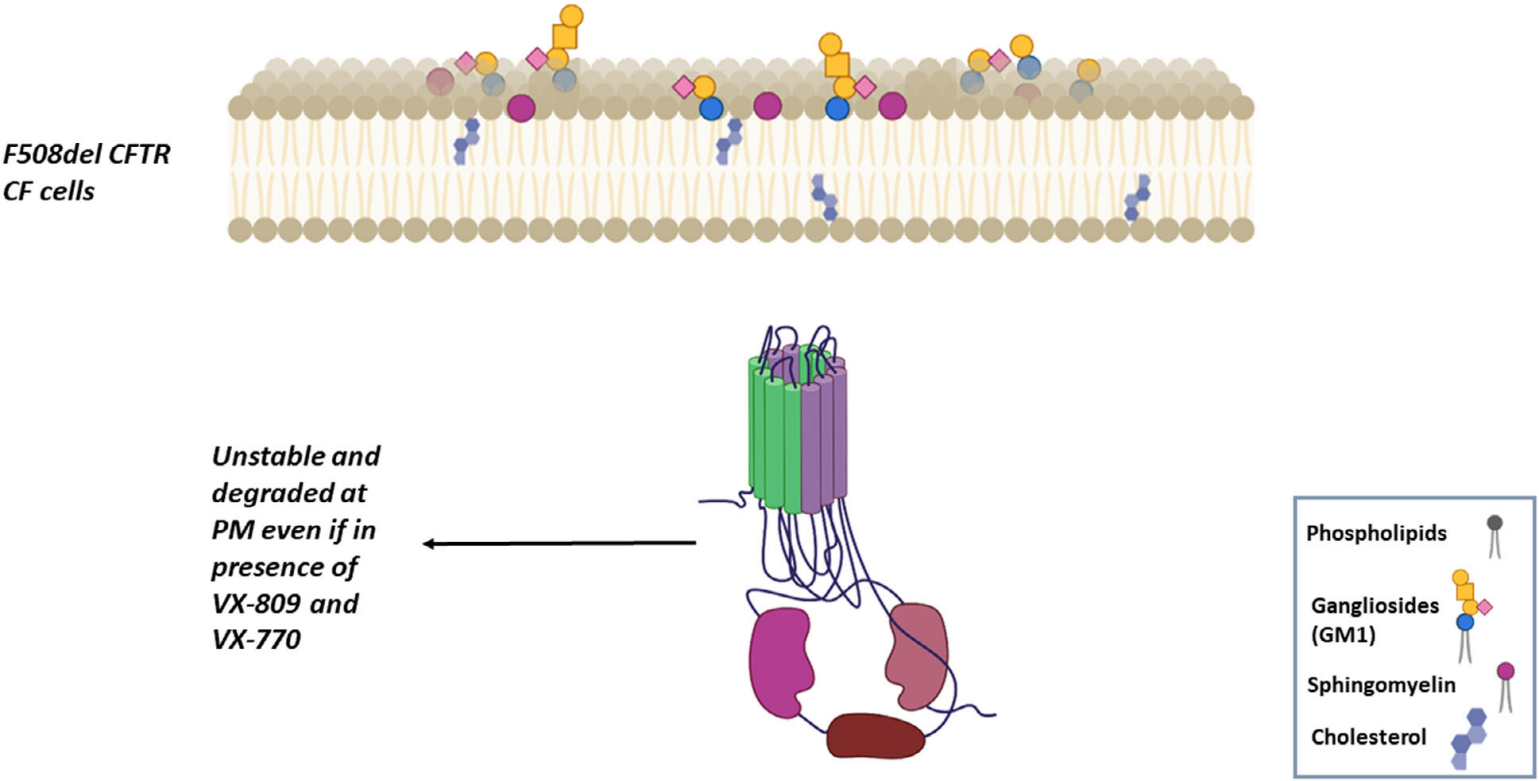
Schematic representation of SL modifications in the PM of HBE cells carrying the F508del and their effect on PM instability of the mutated CFTR rescued by the CFTR modulators.

As mentioned above, in HBE cells the lack of CFTR in the PM due to F508del mutation leads to a reduction in the levels of ganglioside GM1. Treatment with the corrector VX‐809 and/or the potentiator VX‐770 further reduces the level of GM1‐exacerbating modifications in a lipid essential for CFTR function and stability. In fact, an improper lipid environment at the PM might not favor the correction of the F508del mutated channel exerted by modulators, due to protein instability [43], as depicted in Fig. 3.

The protein complex of CFTR, including its scaffolding proteins, NHERF1, and ezrin, organized in the specific lipid environment, plays an important role in the regulation of CFTR function. Ezrin is an A‐kinase anchoring protein that tethers protein kinase A in the proximity of CFTR, allowing cAMP‐dependent control of chloride efflux [60, 61, 62]. Interestingly, the cellular localization of both NHERF1 and phosphorylated Ezrin differs between airway cells expressing WT‐CFTR and F508del‐CFTR, since while normally localized in the apical region, in CF they are also diffusively present in the cytosol [63]. Accumulating evidence suggests that membrane lipids, and especially cholesterol, are directly involved in the complex mechanism regulating the NHERF1–CFTR interaction. Indeed, the PDZ domain of NHERF‐1 specifically binds cholesterol by the cholesterol recognition amino acid consensus motif. Disruption of the cholesterol‐binding activity of NHERF‐1 abolishes its colocalization with CFTR protein and reduces channel activity [64]. The effect of GM1 in organizing the proper PM microenvironment of CFTR could reside also in its effect on the localization of cholesterol in the PM. In particular, in the presence of GM1, the cholesterol moves from the outer leaflet to the inner leaflet of the PM, and here, it can promote the formation of the NHERF‐1‐CFTR complex [65].

AC Kirsten and colleagues found that HBE cells expressing WT‐CFTR form larger lipid domains than cells of the same type that express F508del‐CFTR. Treatment of the latter with the correctors VX‐445 and VX‐661 increased the size of lipid domains, suggesting that the formation of membrane microdomains is inhibited upon reduced CFTR expression at the cell surface [66]. The mechanism by which CFTR modulates raft size remains to be determined; however, it has been shown in different models and biological membranes that the presence and abnormal levels of cholesterol [67] and ω‐3 polyunsaturated fatty acids [68] are relevant in influencing raft stability [69].

Moreover, ceramide levels are important to determine the proper organization of lipid rafts. Even if the increase in the ceramide content in the PM of cultured CF bronchial epithelial cells depends on the cell model, it is clear that its level increases upon bacterial infections. Ceramide forms large platforms at the cell surface from which cholesterol is partially excluded. For this reason, the formation of large ceramide‐rich platforms in response to pathological stimuli [58] seems to negatively affect CFTR function, even if contradictory data are available so far [66]. However, in another study, Abu‐Arish *et al*. suggested that clustering of CFTR enables the protein to enter long‐lived ceramide‐rich platforms when cells are stimulated by thapsigargin, a specific inhibitor of the sarco/endoplasmic reticulum Ca^2+^‐ATPase pump [57], which promotes CFTR accumulation and may also facilitate functional interactions with other proteins [70]. An explanation may be found in the evaluation of the molecular species of ceramide forming the lipid rafts. Indeed, the tight packing induced by ceramides with very long acyl chains (C24:0), such as in cells expressing WT‐CFTR, can interdigitate from both leaflets of the PM [66] promoting CFTR stability. On the contrary, an environment enriched in ceramides with short acyl chains, like in CF cells, might form platforms that may adversely affect CFTR stability due to high fluidity.

Taken together, these findings clearly highlight the important contribution of lipids to the stability and function of CFTR and that restoration of lipid rafts in CF bronchial cells represents a relevant strategy to stabilize the F508del CFTR rescued by different modulators.

## Interfering with SL metabolism ameliorates the phenotype of CF lung disease

It is clear that in CF, the lack of CFTR is associated with a general impairment of SL metabolism resulting in important alterations in their pattern at the PM level. SL are key players in the organization of the structure and architecture of the membrane which, in turn, affects the stability of associated proteins, as well as cell signaling transduction. Therefore, modifications in the SL pattern could affect cellular homeostasis, and thus, there is interest as to whether restoring SL composition to physiological conditions could have a beneficial effect on the different pathological aspects of CF.

Considering the number of studies related to the involvement of Cer in CF, several approaches are based on its reduction, mainly at the level of bronchial epithelial cells. As the production of Cer occurs through three different metabolic means (*de novo* biosynthesis, complex SL catabolism, and sphingosine recycling), it is possible to reduce its levels by modulating these pathways.

Myriocin is a specific inhibitor of the *de novo* biosynthetic pathway of SL acting directly on the serine palmitoyltransferase, the first enzyme responsible for the synthesis of the sphingoid base of Cer [71]. Since this drug acts by blocking the formation of Cer, myriocin does not selectively reduce Cer levels, but affects the entire pattern of cell SL. However, it does not completely inhibit the synthesis of SL, reducing their level to zero, as it acts only on the *de novo* biosynthetic pathway. Therefore, sphingosine derived from the lysosomal catabolism of complex SL remains unaltered and allows, at least partially, sustained SL production. Moreover, the impairment of SL metabolism by myriocin in bronchial epithelial cells facilitates the activation of a stress response that promotes the nuclear translocation of the transcription factor EB (TFEB). TFEB then induces activation of transcription of several genes that encode for proteins responsible for lipid oxidation and autophagy induction [72]. This results in an overall decrease in CF hyperinflammation as a consequence of reduced expression of pro‐inflammatory cytokines, and in an improvement in the defensive response to infections due to an increase in xenophagy activation [19, 73] that is known to be downregulated in CF [74]. Indeed, it was shown that in monocytes isolated from CF individuals carrying biallelic F508del *CFTR* mutation infected with *Aspergillus fumigatus*, the use of myriocin and the consequent rebalance of the SL composition led to certain modifications. The drug alters the expression of genes involved in inflammation, response to infections, lipid metabolism, and autophagy, and, in parallel, upregulates the expression of several proteins associated with the histone family acting as antimicrobial agents [71, 75].

Another interesting approach to reduce Cer levels is based on the use of amitriptyline, an inhibitor of the acid sphingomyelinase currently used in the clinic for the treatment of major depression [76]. Blocking the activity of acid sphingomyelinase in CF bronchial epithelial cells prevents the formation of PM platforms enriched in Cer, leading to a reduction in the inflammatory response [77]. Moreover, studies performed on different CF mouse models revealed that treatment with amitriptyline reduces pulmonary inflammation, preventing and protecting from infections [18, 78]. The data derived from clinical trials on adult CF patients are also encouraging as no severe side effects of amitriptyline were observed in a second phase IIb study, where amitriptyline was administered orally to 29 patients at a dosage of 25 mg twice a day for 28 days. The study showed that the absolute forced expiratory volume in the first second was 7.2% higher in CF individuals treated with amitriptyline than in the placebo group. One‐year follow‐up data of 20 CF individuals taking amitriptyline showed that the mean lung function was 13.1 ± 10.6% higher in the amitriptyline‐treated group than in the control group [79]. Likewise, in 12 of them, mean lung functions were 14.7 ± 18.9% and 19.1 ± 21.7% higher 2 and 3 years after the amitriptyline treatment, respectively. Similar data, with less adverse effects, were obtained in CF mouse models using inhibitors of the acid sphingomyelinase other than amitriptyline, such as trimipramine, desipramine, chlorprothixene, fluoxetine, amlodipine, or sertraline [80].

To reduce Cer content, instead of using sphingomyelinase inhibitors, it is possible to inhibit the enzymes involved in the catabolism of GSL. Indeed, it was shown that treatment with miglustat, a drug used to treat patients affected by Gaucher disease, has a beneficial effect both on inflammation and infection of CF cells, at least *in vitro* [81]. Miglustat is a potent inhibitor of the nonlysosomal β‐glucosylceramidase, an enzyme able to convert GlcCer into glucose and Cer directly at the PM level [82].

In bronchial cells, it was demonstrated that the use of human recombinant acid ceramidase (rhAC), an enzyme able to catabolize the Cer into fatty acid and sphingosine, diminishes the accumulation of Cer. RhAC was originally developed as an enzyme replacement therapy for patients affected by Farber disease, where loss of function mutations in the acid ceramidase gene lead to the accumulation of Cer. In these patients, the enzyme needs to be administered systemically because it must reach the cells throughout the body and then be targeted to lysosomes to exert its function [83]. For CF individuals, rhAC could be delivered by inhalation. Indeed, administration via inhalation of rhAC in CF mouse lungs has been shown to normalize Cer and sphingosine levels. In addition, since rhAC triggers the local conversion of Cer to fatty acid and sphingosine capable of exerting antimicrobial activity, it prevents infections with mucoid and nonmucoid *P. aeruginosa* strains [21]. These data suggest that rhAC could exert its function directly at the PM level, although it is difficult to assume that an enzyme working at acidic pH can operate at the PM, where the pH is classically considered neutral. However, it has been seen that several enzymes operating at acidic pH can also exert their function at the level of the PM, thanks to their close association with proton pumps, which locally establish the appropriate proton concentration [84].

Since one of the problems with the formation of Cer‐enriched platforms is the disruption of β1‐integrin mediated signaling, treatment with β1‐integrin ligands, such as specific antibodies or arginylglycylaspartic acid, has been shown to be beneficial in CF human lungs [21]. The binding of these ligands to β1‐integrin activates the protein and triggers its internalization from the luminal membrane, even in the presence of high Cer concentrations. In CF mice, the inhalation of arginylglycylaspartic acid peptides and anti‐β1‐integrin antibodies prevented pulmonary infections with mucoid and nonmucoid *P. aeruginosa* strains, also resulting in the resolution of an already existing acute or chronic infection [21].

**Fig. 4:**
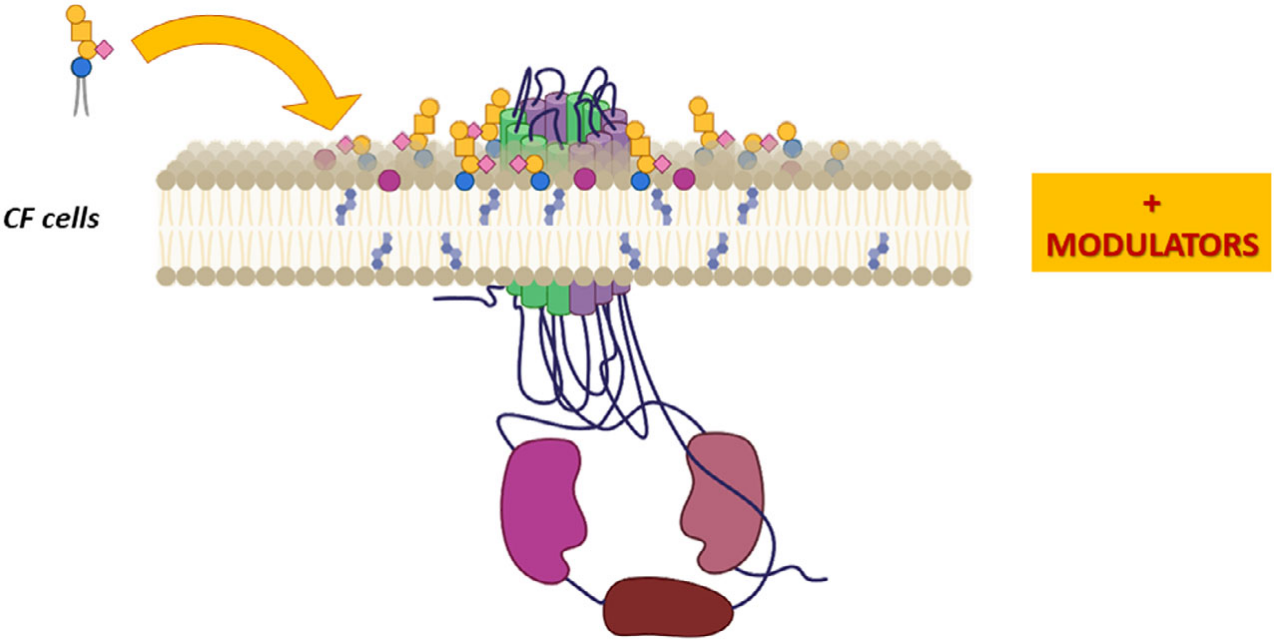
Schematic representation of how the addition of ganglioside GM1 at the PM of bronchial epithelial cells carrying the F508del can ameliorate the PM instability on the mutated CFTR rescued by the CFTR modulators.

The content of one of the important lipid interactors of CFTR, represented by ganglioside GM1, is also reduced in CF bronchial epithelial cells, as mentioned above. Nevertheless, recovery of the proper PM composition by exogenous administration of GM1 results in increased stability and function of CFTR after treatment with correctors and potentiators, and also promotes the recruitment of its scaffolding proteins, NHERF1 and Ezrin [43] (Fig. 4).

Taken together, the evidence reported in the literature suggests that pharmacological modulation of SL metabolism, allowing the SL pattern to return to physiological conditions, may be adjuvant to other therapeutic approaches, and can ameliorate various aspects of CF pathology, with particular regard for lung disease.

## Conclusions

Cystic fibrosis is a widely studied pathology because, even if considered a rare disease, it is quite prevalent in both America and Europe and is one of the most common genetic diseases of Caucasian people. Moreover, the study of CFTR also fascinates the scientific community since over the years, and it has become more and more obvious that this channel is the central node of a very complicated network, where thousands of different actors are involved. It is likely that related studies will allow us to better understand the entire cell physiology as CFTR regulates ionic currents, intracellular trafficking, cell signaling, and metabolism. Among these important processes, a prominent cross‐talk has been described between CFTR and SL as summarized in Table 1. Indeed, CFTR loss of function also affects lipid metabolism, which has significant consequences in the establishment of the phenotype of the pathology. Even if the impairment of SL composition is only of recent interest, over 160 papers to date (PubMed sources) highlight the importance of lipids in CF. Moreover, many studies suggest that lipids, and especially SL, may be important players that could be targeted through therapeutic approaches aimed at rescuing the mutated protein. In particular, in the era of CFTR modulators, the modifications of the SL pattern could represent an important adjuvant approach to further stabilize the rescued mutated channel. The main point is how to modify the sphingolipid pattern in order to restore the composition found in cells carrying WT‐CFTR. In this direction, the repositioning of some available drugs could represent several options, such as the case of the amitriptyline and myriocin cited in the paragraph above. Another possibility consists in the administration of recombinant enzymes, but in our opinion, this is too complicated to be applied. One option, due to the great stability of these molecules, is administration of SL. For instance, the ganglioside GM1 is currently used in some countries for the treatment of peripheral neuropathies, and this opens a real possibility for its use in CF. In CF, almost all the knowledge of SL is related to the F508del mutation and was obtained by analyzing the lungs of individuals with CF or using *in vitro* models, such as primary bronchial epithelial cells differentiated at ALI or immortalized cell lines. However, we know of more than 2000 mutations causing the disease, and know that CF is a systemic disorder with a severe impairment of other organs beyond the lungs. For these reasons, we are seeing only the tip of the iceberg so far, and a huge amount of work needs to be conducted in the future to dissect the true role of SL in the development of clinical phenotypes in CF. Can sphingolipids act as modifying molecules in CF? This may be one of the new open questions to be answered.

**Table 1:** Changes in the SL content of CF cells.

	General ceramide content	Molecular species of ceramide (C16, C18, C20)	Molecular species of ceramide (C24)	SM	Sphingosine	Glycosphingolipids				
						GlcCer	LacCer	GM3	GM1	Asialo‐GM1
Increase	✖	✖				✖	✖	✖		✖
Decrease			✖	✖	✖				✖	

## Conflict of interest

The authors declare no conflict of interest.

## Author contributions

MA and DD conceived and coordinated the paper, and they wrote the introduction, abstract, and conclusions. NL wrote the paragraph ‘CFTR deficiency and its correlation with the sphingolipid pattern’. RB wrote the paragraph ‘Sphingolipids’. AP wrote the paragraph ‘Cystic fibrosis’. GL wrote the paragraph ‘Sphingolipids as interactors of CFTR’. AT wrote the paragraph ‘Interfering with the SL metabolism ameliorates the phenotype of CF lung disease’.
